# Mononuclear phagocyte system blockade improves therapeutic exosome delivery to the myocardium

**DOI:** 10.7150/thno.38198

**Published:** 2020-01-01

**Authors:** Zhuo Wan, Lianbi Zhao, Fan Lu, Xiaotong Gao, Yan Dong, Yingxin Zhao, Mengying Wei, Guodong Yang, Changyang Xing, Li Liu

**Affiliations:** 1 Department of Hematology, Tangdu Hospital, Fourth Military Medical University, Xi'an, 710038, People's Republic of China.; 2 Department of Ultrasound Medicine, Tangdu Hospital, Fourth Military Medical University, Xi'an, 710038, People's Republic of China.; 3 State Key Laboratory of Cancer Biology, Department of Biochemistry and Molecular Biology, Fourth Military Medical University, Xi'an, 710032, People's Republic of China.

**Keywords:** Exosomes, targeted delivery, blocking, clathrin, doxorubicin-induced cardiotoxicity

## Abstract

**Rationale:** Exosomes are emerging as a promising drug delivery carrier. However, rapid uptake of exosomes by the mononuclear phagocyte system (MPS) remains an obstacle for drug delivery into other targeted organs, including the heart. We hypothesized that prior blocking of uptake of exosomes by the MPS would improve their delivery to the targeted organs.

**Methods:** Exosomes were isolated from the cell culture medium. Fluorescence-labeled exosomes were tracked *in vitro* and *in vivo* by fluorescence imaging. The expression of clathrin heavy chain (Cltc), cavolin1, Pak1 and Rhoa, known genes for endocytosis, were profiled in various cell lines and organs by qPCR. The knockdown efficiency of siRNA against Cltc was analyzed by Western blotting. Exosome^control^ and exosome^blocking^ were constructed by encapsulating isolated exosomes with siControl or siClathrin via electroporation, while exosome^therapeutic^ was constructed by encapsulating isolated exosomes with miR-21a. Doxorubicin-induced cardiotoxicity model was used to verify the therapeutic efficiency of the exosome-based miR-21a delivery by echocardiography.

**Results:** Exosomes were preferentially accumulated in the liver and spleen, mainly due to the presence of abundant macrophages. Besides the well-known phagocytic effect, efficient endocytosis also contributes to the uptake of exosomes by macrophages. Cltc was found to be highly expressed in the macrophages compared with other endocytosis-associated genes. Accordingly, knockdown of Cltc significantly decreased the uptake of exosomes by macrophages *in vitro* and *in vivo*. Moreover, prior injection of exosome^blocking^ strikingly improved the delivery efficiency of exosomes to organs other than spleen and liver. Consistently, compared with the direct injection of exosome^therapeutic^, prior injection of exosome^blocking^ produced a much better therapeutic effect on cardiac function in the doxorubicin-induced cardiotoxicity mouse model.

**Conclusions:** Prior blocking of endocytosis of exosomes by macrophages with exosome^blocking^ successfully and efficiently improves the distribution of following exosome^therapeutic^ in targeted organs, like the heart. The established two-step exosome delivery strategy (blocking the uptake of exosomes first followed by delivery of therapeutic exosomes) would be a promising method for gene therapy.

## Introduction

Exosomes, which are small membrane vesicles of 30-200 nm in diameter, are secreted by different types of cells and are emerging as a novel mediator of intercellular communication and drug delivery vehicles 1, 2. As naturally derived nanovesicles, exosomes efficiently deliver exogenous RNAs (siRNAs and miRNAs) to target tissues/cells *in vivo* 3. Despite multiple advantages over existing synthetic systems, such as less immunogenicity and higher affinity, exosomes suffer from the drawback of endocytosis by the mononuclear phagocyte system (MPS), such as in the liver and spleen, limiting their distribution in other tissues like heart 4. Due to the high prevalence of heart disease, there is a great demand for improving the cardiomyocyte-specific delivery of exosomes.

Previous studies mostly focused on engineering targeted peptides with improved delivery efficiency specific to cardiomyocytes 5, 6. However, the efficiency is compromised as the liver and spleen remain the primary target organs for the engineered exosomes 5. Therefore, reducing the non-specific accumulation of exosomes in the liver and spleen must be circumvented to increase the delivery efficiency of exosomes to the heart and other organs.

The process of endocytosis is divided into phagocytosis and pinocytosis 7. Phagocytosis involves internalization of relatively large (> 500 nm) particles and is typically restricted to specialized phagocytes 8. In contrast, pinocytosis is exhibited by all cells and is commonly classified into clathrin-dependent endocytosis (CDE), clathrin-independent endocytosis (CIE), and macropinocytosis (MP) 9, 10. Clathrin is a protein that plays a major role in the formation of coated vesicles. It forms a triskelion shape composed of three clathrin heavy chains and three light chains, which spontaneously assemble into a basket-like lattice to drive the budding process of endocytosis 11. Clathrin heavy chain 1 (Cltc) is a protein encoded by the CLTC gene. Caveolin-1 is indispensable for the formation of caveolae and accumulates in membrane invaginations, critical in the CIE process 12. P1-activated kinase1 (PAK1), which plays an important role in MP, is a 62-68 kDa serine/threonine kinase identified as a target of the Rac and Cdc42 (GTPase) and regulates the actin-myosin cytoskeleton 13. RhoA is a member of the Rho family of small GTPases and participates in the process of phagocytosis 14. Identification of the key genes accounting for the differences in the endocytosis of exosomes by spleen, liver, and heart would probably shed light on improving heart-targeted delivery efficiency.

In this study, we found that Cltc plays a significant role in mediating endocytosis of exosomes in the liver and spleen. By employing the doxorubicin-induced cardiac deficiency model, we confirmed that prior injection of exosome^blocking^ significantly and specifically blocks the endocytosis of exosomes in the liver and spleen and allows the subsequent injection of exosome^therapeutic^ to prevent the myocardial damage from doxorubicin efficiently.

## Materials and Methods

### Mice husbandry

Male C57/B6 mice (6-8 weeks old) were purchased from the Experimental Animal Center of Fourth Military Medical University. Mice were fed under SPF conditions. The experimental procedures were performed in accordance with the guidelines approved by the Institutional Animal Experiment Administration Committee of the Fourth Military Medical University.

### Isolation of neonatal rat cardiomyocytes

Neonatal rats (1-2 d postnatal) were purchased from Experimental Animal Center of Fourth Military Medical University. Neonatal rats were dipped in 75% ethanol for 10 s twice after sacrifice. The hearts of neonatal rats were harvested by sterile ophthalmic scissors and cleaned in phosphate-buffered saline (PBS) to remove excess blood clots. The heart tissue was cut into pieces and digested with enzymes (a mixture of 0.5% Collagenase I and 0.05% Trypsin). After digestion for 20 min at 37 °C, the mixture was filtered, and the solution was centrifuged at 1000 rpm for 5 min. The supernatant was discarded and the sediment was resuspended in 10 mM BrdU-supplemented DMEM growth medium. After adherence for 2 h, the adherent cells were removed while the supernatant was grown on a new 6-well plate as the cardiomyocyte culture.

### Isolation of primary hepatocytes

Male C57/B6 mice (6-8 weeks old) were purchased from the Experimental Animal Center of Fourth Military Medical University. After sacrifice, mice were dipped in 75% ethanol for 10 s twice. The liver was perfused by 50 ml sterile perfusion fluid via postcava to remove red blood cells and granulocytes. Subsequently, the liver tissue was cut into pieces and digested with 0.1 mg/ml Collagenase IV for 1 h at 37 °C, and the mixture was filtered through a 200 mesh filter. The supernatant was centrifuged for 5 min at 1000rpm, and the sediment was resuspended in the culture medium.

### Cell culture

RAW264.7 and HUVEC cells were obtained from ATCC. All cells were cultured in H-DMEM (Hyclone, US) supplemented with 10% fetal bovine serum (Exocell, China), 1% antibiotics (Hyclone, US) at 37 °C with 5% CO2. Cells were passaged every three days or when confluent.

### qPCR analysis of mRNA and miRNA expression

Total RNA was isolated from tissues, cells, or exosomes using Trizol (Invitrogen) according to the manufacturer's instructions. Reverse-transcription was conducted using PrimeScript First-Strand cDNA Synthesis Kit (Takara, China) for mRNA. miRNA was reverse-transcribed to cDNA by using miRcute First-Strand cDNA Synthesis Kit (Tiangen Biotech, China). Gene expression was analyzed using PrimeScript RT Master Mix (Roche, Switzerland) or miRcute miRNA qPCR Kit (Roche, Switzerland). All PCR reactions were run at least in triplicate, and the target mRNA/miRNA expression was normalized to GAPDH/U6, respectively. Relative expression was calculated by normalizing to the control samples. The primer sequences are listed in Table S1.

### Exosome isolation and characterization

HEK 293T cells were used as the exosome donor cells in the study. Exosomes were isolated by ultracentrifugation or using the Exoquick^-TC^ kit. For ultracentrifugation, cell culture supernatants were collected after 48 h of culture, followed by centrifugation at 300 g for 5 min to remove dead cells. The supernatants were first centrifuged at 3,000 g for 25 min to eliminate cellular debris, then additionally centrifuged at 100,000 g for 3 h. The sediment was resuspended in PBS, and the mixture was additionally centrifuged at 100,000 g for 1 h to obtain relatively pure exosomes. For isolation of exosomes using Exoquick^-TC^, cell culture supernatants were processed following the manufacturer's protocol. The exosomes were resuspended in PBS and stored at -80 °C till use. The isolated exosomes were diluted to 1 mg/ml, and the size distribution was analyzed by NanoSight (Malvern, UK). The morphology of isolated exosomes was observed by electron microscopy (JEM-2000EX TEM, Japan).

### Doxorubicin treatment

Mice were injected intraperitoneally with DOX (Yuanye biotechnology, China) diluted in PBS at 5 mg/kg/week for four consecutive weeks. At the end of the experiment, the mice were subjected to echocardiography examination and/or histology analysis.

### siRNA loading into exosomes

Exosomes at a protein concentration of 1 mg/ml (BCA Protein Assay Kit, Thermo Scientific) were electroporated with 1 OD siRNA/ miRNA mimics (GenePharma, Shanghai) at 700 V/150 mF in 0.4 cm wide electroporation cuvettes. Sequences for miRNAs/siRNAs are listed in Table S2. After electroporation, exosomes were treated with RNase to remove miRNAs that may be bound to the membrane of the exosomes. Subsequently, exosomes were washed with cold PBS three times and isolated by ultracentrifugation or using Exoquick^-TC^ kit by centrifugation at 12,000 g for 30 min to remove the unincorporated free nucleic acids. The exosome suspension was further vibrated to dissociate the possible aggregates.

### *In vitro* and *in vivo* fluorescence tracing of exosomes

Exosomes (1 µg/µL) were labeled with DiI or DiR by incubating with the dye (1 mM) at the ratio of (500:1 in volume) for 30 min, followed by exosome isolation as described above.

For *in vitro* tracing of exosomes in macrophages, RAW264.7 cells with different treatments were incubated with DiI-labeled exosomes for 3 h. The cells were then washed with PBS three times and fixed with 4% paraformaldehyde for 10 min and again washed with PBS twice. The cell nuclei were counter-stained with Hoechst33342 (1:1,000, Beyotime Biotechnology) for 10 min at 37 °C. At the end of the experiment, the cells were washed with sodium acetate solution (to remove the nonspecific adhesion) and observed using a Nikon A1 Spectral Confocal Microscope (Nikon, Japan).

For the *ex vivo* fluorescence tracing of exosomes, control mice or mice with indicated treatments were additionally injected with 200 μg of DiR-labeled exosomes via the tail vein. The localization of the exosomes in different organs was detected by imaging using the IVIS^®^ Lumina II *in vivo* imaging system (PerkinElmer, Thermo Fisher, US).

### Animal treatment of exosomes

To block the endocytosis function of the liver and spleen, the mice were intravenously injected with siClathrin or siControl loaded exosomes (0.5 OD siRNA/200 μg exosomes per mouse) 3 days before DOX treatment. Then, the mice were intravenously injected with control or miR-21a-5p mimic-loaded exosomes (0.5 OD mimics/200μg exosomes) one day before DOX treatment. The exosome injection procedure was repeated every week during the 4 weeks of DOX treatment.

### Immunofluorescence

To view the exosome cellular uptake by macrophages in the liver tissue, the injected exosomes were labeled with DiI as described above. The cells with DiI-labeled exosome uptake were thus DiI-positive. For the immunofluorescence staining of the tissue, sections of 8 μm thickness were prepared using a cryostat. After incubation with 5% bovine serum albumin (BSA) for 1 h, the sections were incubated with primary antibody (anti-F4/80, 1:500, Abcam, USA, ab6640; anti-cTnT, 1:500, Abcam, USA, ab8295) overnight at 4 °C in a wet, dark box. Subsequently, the sections were incubated with the secondary antibody (AlexaFluor 488- rat anti-mouse, 1:800, Invitrogen) for 1 h at room temperature. Cell nuclei were stained with Hoechst 33342. The sections were washed with PBS and then observed with a Nikon A1 Spectral Confocal Microscope (Nikon, Japan).

### Western blotting

Isolated cells and exosomes were subjected to RIPA lysis buffer (Beyotime Biotechnology, China) supplemented with the Protease Inhibitor Cocktail (Roche). Purified proteins were separated in 6%, 10%, or 12% SDS-PAGE (120 V for stacking gel and 160 V for separation gel) and then transferred to a nitrocellulose membrane in an ice bath. The nitrocellulose membrane was blocked with 5% bovine serum albumin for 1 h and then incubated overnight with primary antibodies at 4 °C. Antibodies used were mouse anti-CD63 (Abcam, ab59479), rabbit anti-CD9 (Abcam, ab92726), mouse anti-TSG101 (Santa, sc-7964), rabbit anti-GM130 (Abcam, ab30637), rabbit anti-Cltc (Cell Signaling Technology, #4796), rabbit anti-GAPDH (Abcam, ab181602). The membrane was then incubated with secondary antibodies (rat anti-mouse (Abcam, ab99632), mouse anti-rabbit (Abcam, ab99702)) for 1 h at room temperature and visualized using the ECL Prime Western Blotting Detection Reagent (GE Healthcare, Buckinghamshire UK).

### Histology and Masson staining

The mice were intraperitoneally anesthetized with 120 mg/kg body weight of ketamine and 24 mg/kg body weight xylazine in 0.9% sodium chloride. After complete anesthesia, the mouse thorax was opened and perfused with 4% paraformaldehyde from the apex of the mouse. After perfusion, the heart, liver, and spleen of mice were removed and soaked in 4% paraformaldehyde for 24 h. The tissues were placed in the embedding box and rinsed with running water for 30 minutes. After dehydration, transparency, waxing, embedding, sectioning, and spreading, staining was performed with hematoxylin and eosin (Beyotime, China). The heart sections were also subjected to Masson staining using the Masson Trichrome Staining Kit according to the manufacturer's instructions (Solarbio, China).

### Echocardiography

For echocardiographic measurement, mice were anesthetized with 2.5-3.0% isoflurane. Transthoracic echocardiography was conducted using the Vevo 2100 high-resolution imaging system (VisualSonic, Canada) equipped with a 13-24 MHz transducer. Two-dimensional short-axis M-mode echocardiography was conducted at the level of the mid-papillary muscle. Left ventricular ejection fraction (LVEF) and fractional shorting values were calculated. All M-mode tracing evaluation parameters were averaged from five cardiac cycles. Investigators were blinded to the identity of animals.

### TUNEL staining

The heart tissue was embedded with OCT and sectioned at a thickness of 8 μm. The remaining steps were carried out according to the instruction manual of the TUNEL staining kit (Roche). The nuclei were counter-stained with Hoechst. After staining, the cardiomyocyte apoptosis was observed by confocal microscopy (Nikon, Japan).

### Statistics

The data were expressed as mean ± SEM and analyzed by one-way ANOVA analysis or t-test. Differences were considered significant at p < 0.05. All statistical analyses were conducted by SPSS 21.0 or GraphPad Prism7.

## Results

### 1. Predominant uptake of exosomes in the liver and spleen by the macrophages

**Figure 1:**
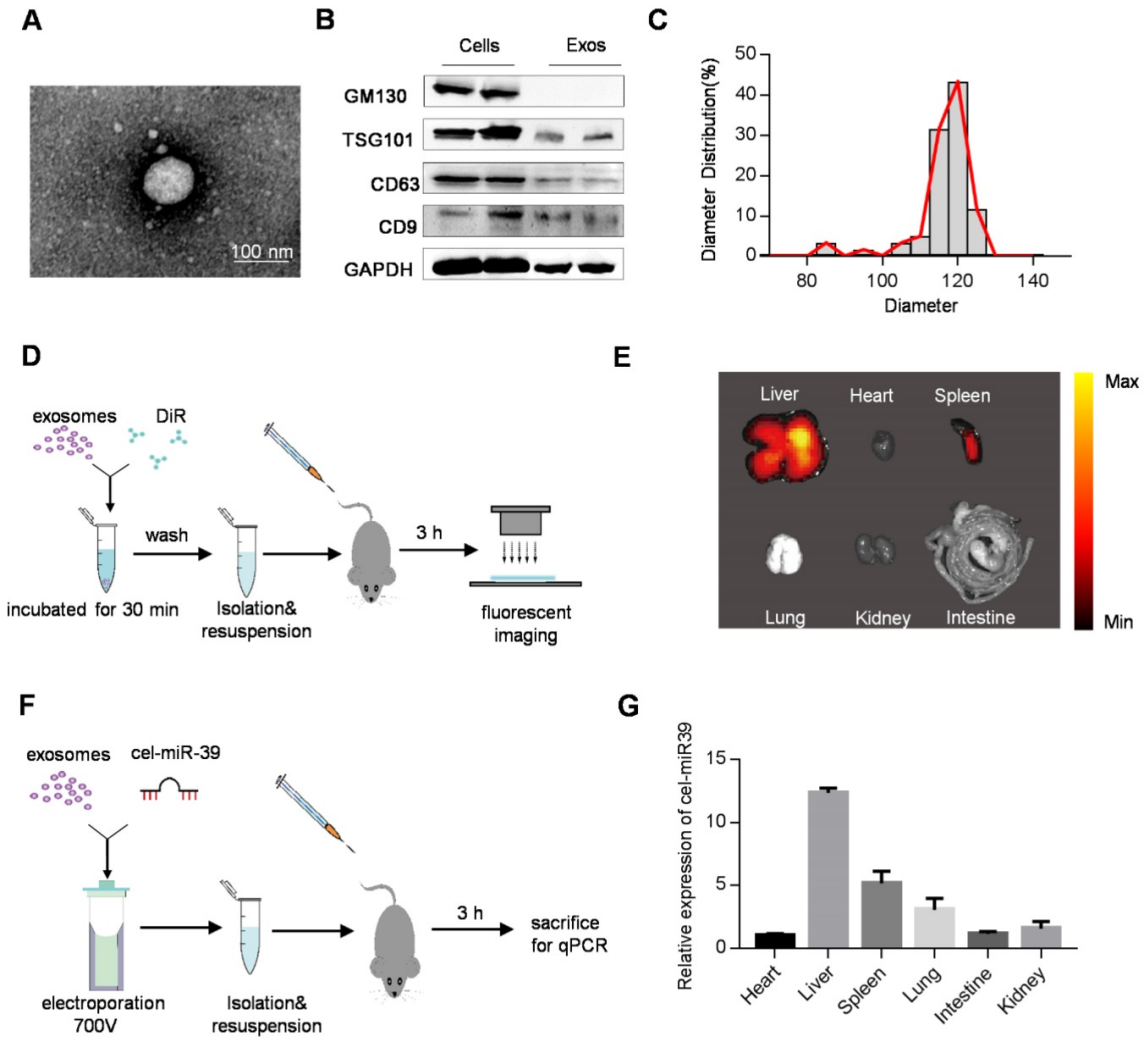
** Distribution of exosomes in mice**. (A) Representative transmission electron microscope image of 293T cell-derived exosomes. Scale bar=100 nm. (B) Western blot analysis of exosomal markers (TSG101, CD63, CD9) and cellular markers (GM130) in the isolated exosomes and the cells. GAPDH served as internal control. (C) Diameter distribution of exosomes analyzed by Nanosight. (D) Schematic diagram of the experimental procedure. Mice were injected with 100 μg of DiR-labeled exosomes to monitor the distribution of exosomes. (E) Representative *ex vivo* fluorescent images of various organs in mice injected with DiR-labeled exosomes. n=5 mice. (F) Schematic diagram of the experimental procedure. Mice were injected with cel-miR-39-loaded exosomes to monitor the distribution of exosomes. (G) qPCR analysis of expression of cel-miR-39 in various organs in mice. U6 served as an internal control. The expression of cel-miR-39 in various organs was normalized to the heart. Data are expressed as mean ± SEM of five independent biological samples.

In this study, we used HEK293T cell-derived exosomes, which displayed the typical exosome morphology as revealed by transmission electron microscopy (Figure 1A). Western blot analysis showed that the isolated exosomes expressed exosomal markers such as TSG101, CD63, and CD9, but did not express the cell-specific marker GM130 (Figure 1B). As shown in Figure 1C, NanoSight analysis revealed that the diameters of these vesicles ranged from 30 to 150 nm. These data confirmed the exosome identity of the isolated vesicles. Although most of the extracellular vesicles consisted of exosomes, a small part of other subpopulations could also be pelleted by the isolation method.

To track the efficient distribution of exosomes to the target organ, which is a prerequisite for drug delivery, we injected DiR-labeled exosomes (Figure S1A) into mice via the tail vein (Figure 1D). As expected, *ex vivo* fluorescent imaging showed that the liver and spleen were the dominant targets of exosomes with the strongest fluorescence intensity (Figure 1E). To further confirm the results, mice were injected with cel-miR39-loaded exosomes (Figure 1F), as cel-miR-39 has no homolog in mice 15, 16. qPCR analysis of cel-miR-39 abundance in different organs showed that most exosomes were internalized by the liver and spleen, with only a small fraction taken up by the heart (Figure 1G).

**Figure 2:**
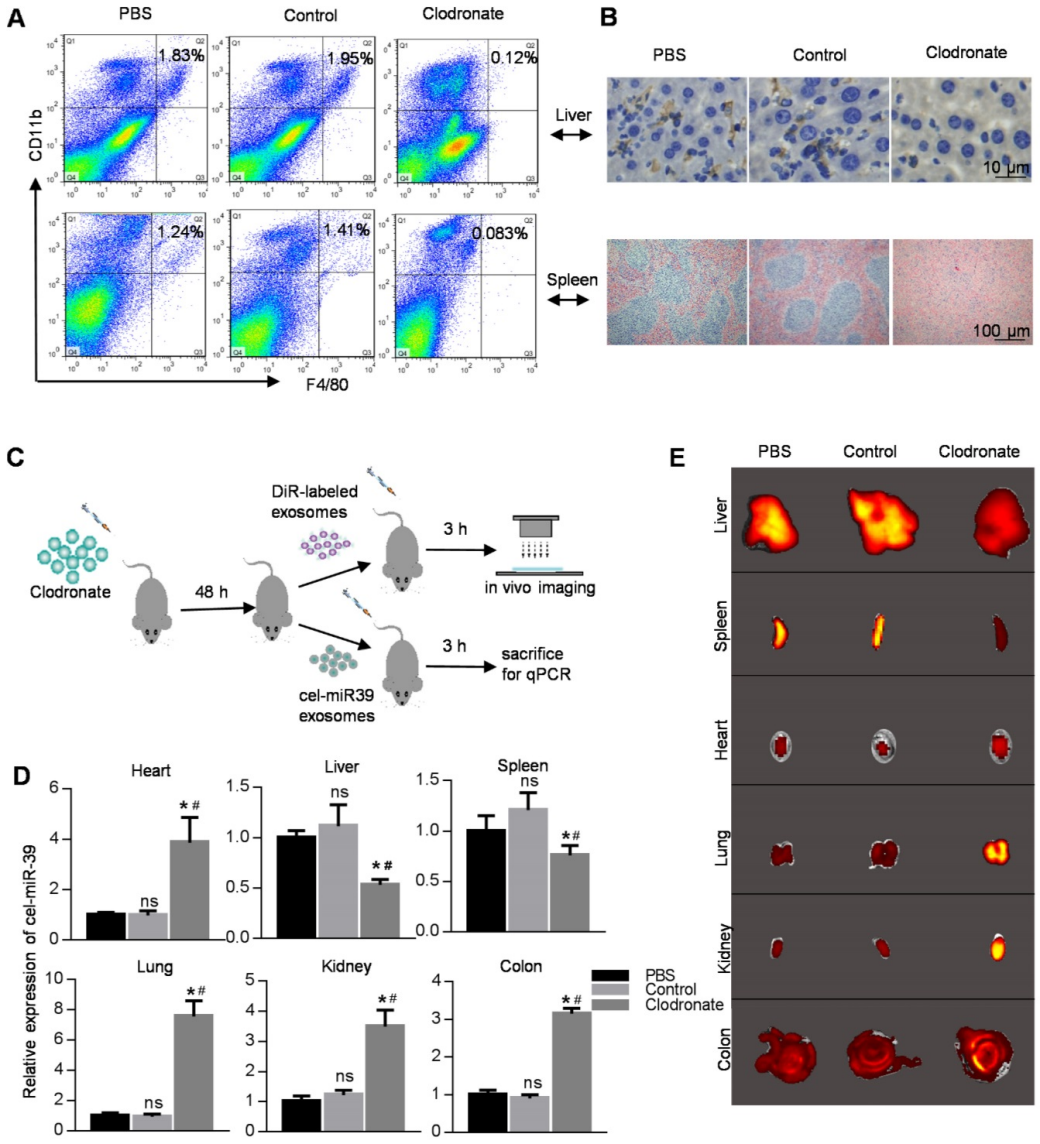
** Distribution of exosomes in macrophage-depleted mice.** (A) FACS analysis of CD11b-APC versus F4/80-FITC in cells from liver and spleen. Mice were treated with PBS/liposomes/Clodronate liposomes (0.1 ml/10 g) for 48 h. n=5 mice per group. (B) Histology analysis of macrophages from liver and spleen. Representative immunohistochemical images of F4/80 in the liver (top) and spleen (bottom). Mice were treated with PBS/liposomes/Clodronate liposomes (0.1 ml/10g) for 48 h. n=5 mice per group. (C) Schematic diagram of the experimental procedure. Mice injected with PBS/liposomes/Clodronate liposomes were additionally injected with DiR-labeled exosomes/cel-miR-39 loaded exosomes, followed by qPCR and *ex vivo* imaging in various organs. (D) qPCR analysis of expression of cel-miR-39 in various organs in mice treated with PBS/liposomes/Clodronate liposomes. U6 served as an internal control. Data are expressed as mean ± SEM of five independent biological samples. *, p < 0.05 compared with PBS group; ns, not significant compared with PBS group; #, p < 0.05 compared with control groups. (E) Representative fluorescent images showing the distribution of DiR-labeled exosomes in various organs in mice treated with PBS/liposomes/Clodronate liposomes. n=5 mice per group.

The macrophage population is significantly high in both spleen and liver, and their phagocytic activity is very likely the reason for the dominant localization of exosomes in these organs. We, therefore, used clodronate liposomes to eliminate macrophages in mice. FACS analysis, immunohistochemistry, and HE staining showed that almost all macrophages were eradicated in the clodronate group (Figure 2A, B). After macrophage depletion, we injected DiR-labeled exosomes or cel-miR-39-loaded exosomes into mice (Figure 2C). qPCR analysis of the cel-miR-39 or fluorescence imaging of DiR-labeled exosomes revealed that macrophage depletion significantly decreased the exosome localization in the liver and spleen with a simultaneously increased level of the exosomes in the blood (Figure 2D, 2E, Figure S1B, S1C). Consistently, the exosomes in other organs, such as heart, colon, and lung, were increased (Figure 2D, 2E, Figure S1B, S1C). Confocal images were consistent with the results of fluorescence imaging (Figure S1D).

### 2. Cltc plays an important role in exosome endocytosis by macrophages

**Figure 3:**
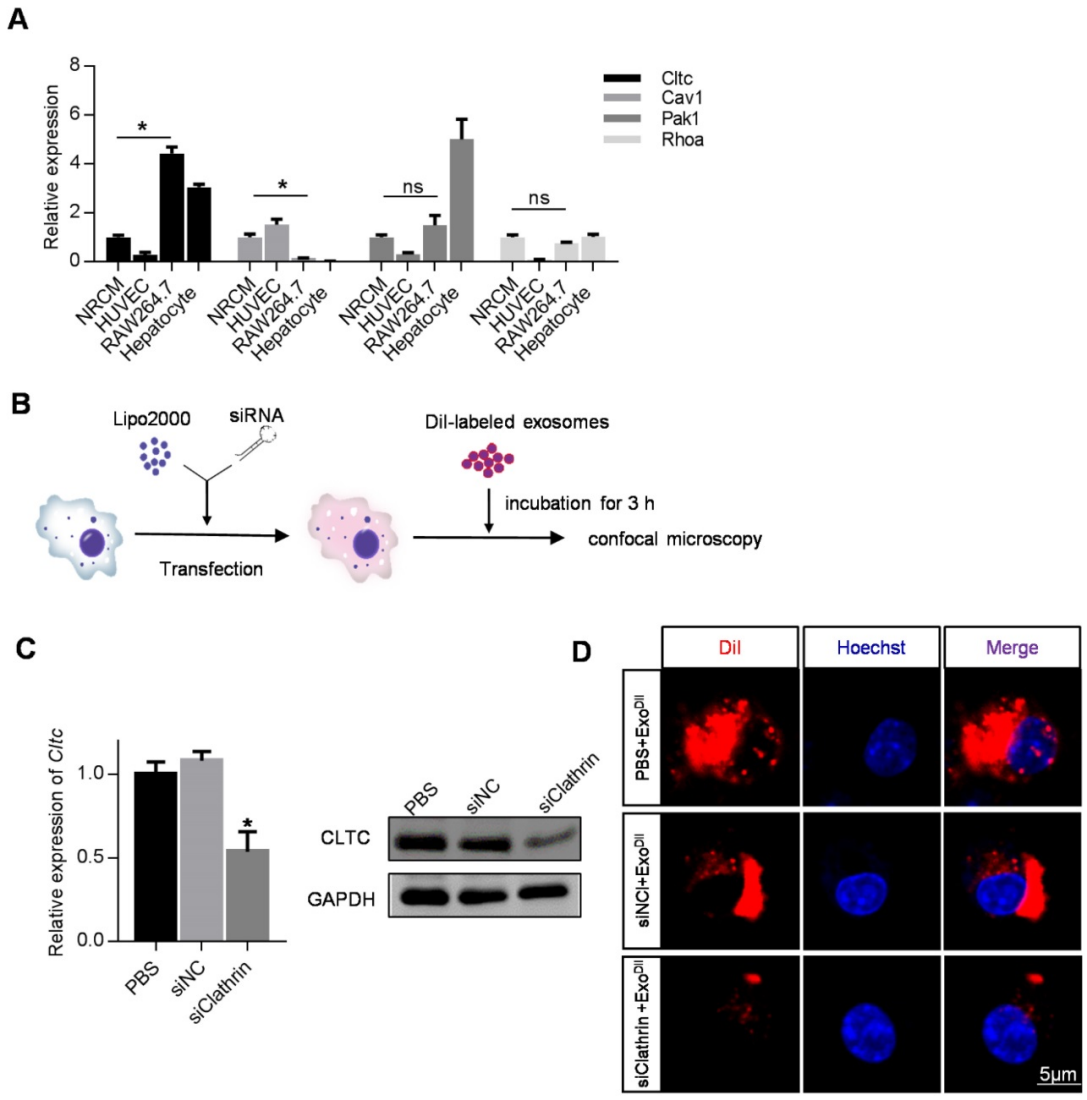
** Cltc plays an important role in exosome endocytosis**. (A) qPCR analysis of expression of endocytosis-related genes (Cltc, Cav1, Pak1, and RhoA) in RAW264.7, HUVECs, and NRCMS cells, and primary hepatocytes. GAPDH served as an internal control. Data are expressed as mean ± SEM of five independent biological samples. *, p < 0.05; ns, no significance. (B) Schematic diagram of the experimental procedure. DiI-labeled exosomes were added to RAW264.7 cells treated with PBS/siControl/siClathrin to monitor exosome uptake. (C) qPCR and Western blot analysis of Cltc knockdown efficiency in RAW264.7. GAPDH served as an internal control. Data are expressed as mean ± SEM. n=5, *p < 0.05. (D) Representative fluorescence image of the DiI-labeled exosomes (red) in RAW264.7 cells. The nuclei were counter-stained with Hoechst (blue). Scale bar=5 μm. DiI-labeled exosomes (50 ng/ml) were added to RAW264.7 cells and incubated for 3 h before fluorescence microscopy.

Given the above data, we attempted to identify genes responsible for the robust endocytosis of exosomes by macrophages. The expression of Cltc, Cav1, Pak1, and Rhoa in the heart, liver, spleen, lung, kidney, and colon in mice was analyzed by qRT-PCR. Among these candidates, the expression of Cltc was the highest in the liver compared with the other three genes (Figure S2). Cltc expression was also much higher in the liver, compared with that in the heart (Figure S2). To further confirm the differential expression of these candidate genes in macrophages and other cell types, expression in NRCMs (cardiomyocytes), HUVEC (endothelial cells), RAW264.7 (macrophages) and primary hepatocytes was also analyzed. Among the cell types examined, expression of Cltc in RAW264.7 and hepatocytes was found to be much higher than that in NRCMs (Figure 3A). These results indicated that Cltc might play an important role in endocytosis by macrophages and hepatocytes. We thus explored the effects of Cltc knockdown on endocytosis in RAW264.7 cells (Figure 3B). With Cltc significantly knocked down by lipofectamine-mediated transfection (Figure 3C), the uptake of exosomes by RAW264.7 cells was markedly reduced, as seen from the decreased DiI positive signal by fluorescence confocal microscopy (Figure 3D).

### 3. Prior injection of exosome^blocking^ significantly blocks MPS

**Figure 4:**
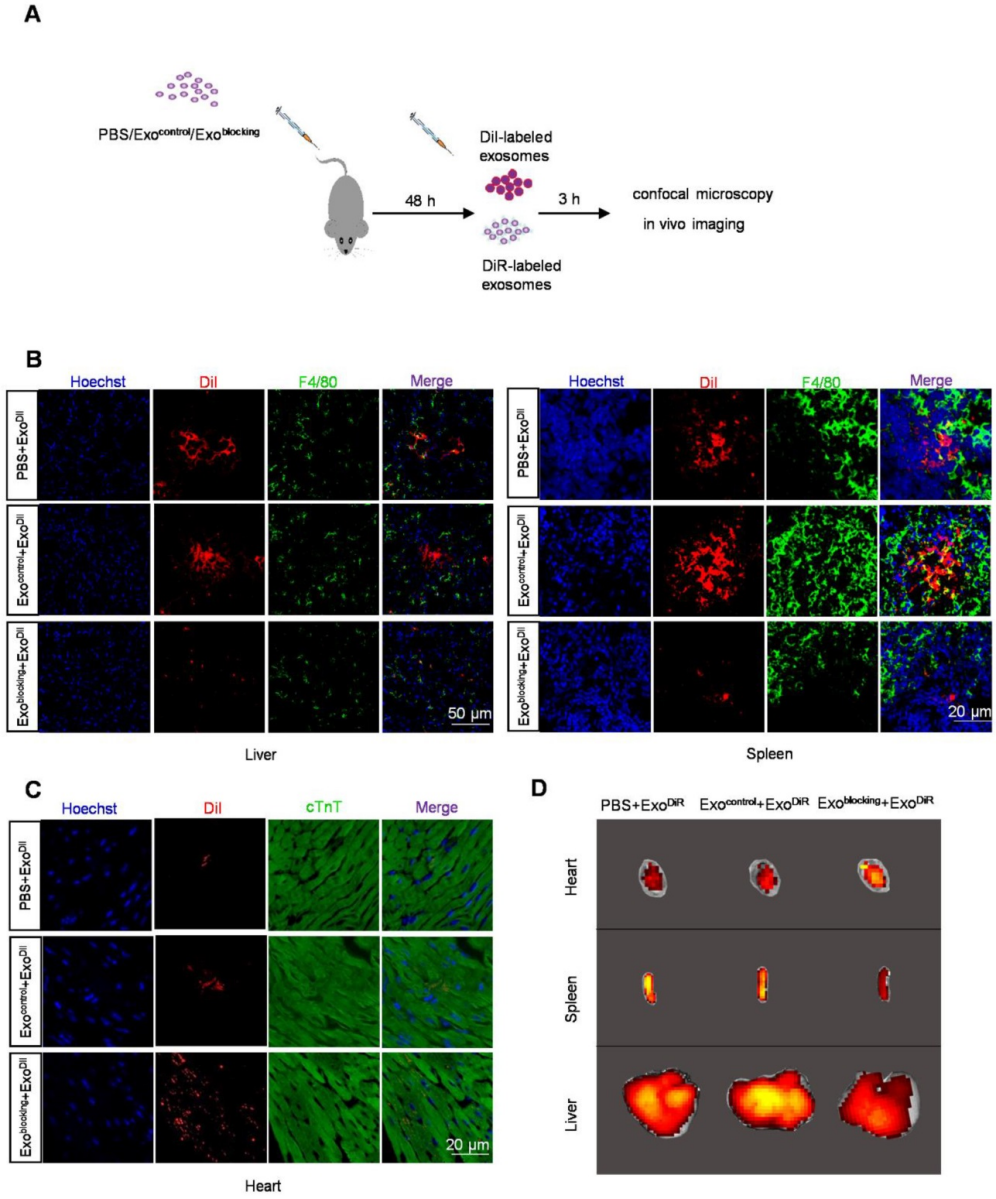
** Pre-treatment with exosome^blocking^ improves the delivery efficiency of exosome^DiI^ to the myocardium.** (A) Schematic diagram of the experiment. Mice pre-injected with PBS/exosome^control^/exosome^blocking^ were injected with DiI-labeled exosomes for tracking. (B) Representative fluorescence images of the DiI-labeled exosomes (red) in the liver and spleen. Macrophages were stained with F4/80 (green) while the nuclei were counter-stained with Hoechst (blue). Scale bar = 50 or 20 μm as indicated. n=5 per group. (C) Representative fluorescence images of the DiI-labeled exosomes (red) in the heart. Cardiomyocytes were stained with cTnT (green) while the nuclei were counter-stained with Hoechst (blue). Scale bar = 50 μm. n=5 per group. (D) Representative fluorescence images showing the localization of DiR-labeled exosomes in various organs in mice treated with PBS/exosome^control^/exosome^blocking^.

The above findings suggested that knockdown of Cltc *in vivo* would probably block the MPS. To knock down Cltc *in vivo*, especially in the liver and spleen, siClathrin was encapsulated into the exosomes (Figure 4A) and the modified exosomes were designated as exosome^blocking^. Similar to siClathrin knockdown, *in vitro* experiments confirmed that exosome^blocking^ significantly inhibited the uptake of exosomes by RAW264.7 cells (Figure S3A-B). For *in vivo* experiments, 48 h after injection of exosome^blocking^, DiI-labeled exosomes (exosome^DiI^) were injected into the mice (Figure 4A). Fluorescence imaging showed that Cltc was efficiently decreased only in the liver and spleen after exosome^blocking^ (Figure S4A-C). Also, prior injection of exosome^blocking^ substantially reduced the localization of exosome^DiI^ injected later in the liver and spleen (Figure 4B). In contrast, prior injection of exosome^blocking^ considerably improved the cardiomyocyte localization of exosome^DiI^ injected later (Figure 4C-D). Similar results were observed in *ex vivo* tracing of the DiR-labeled exosomes (exosome^DiR^) (Figure 4D, Figure S5A). Also, cel-miR-39 tracing experiments independently confirmed a significant reduction in the liver and spleen distribution while improvement in the cardiomyocyte localization of later injected exosomes by prior injection of exosome^blocking^ (Figure S5B). These results demonstrated the successful construction of a safe and convenient system to improve exosome delivery to the heart.

### 4. Prior injection of exosome^blocking^ improves the beneficial effect of therapeutic exosomes in DOX-induced cardiotoxicity model

**Figure 5:**
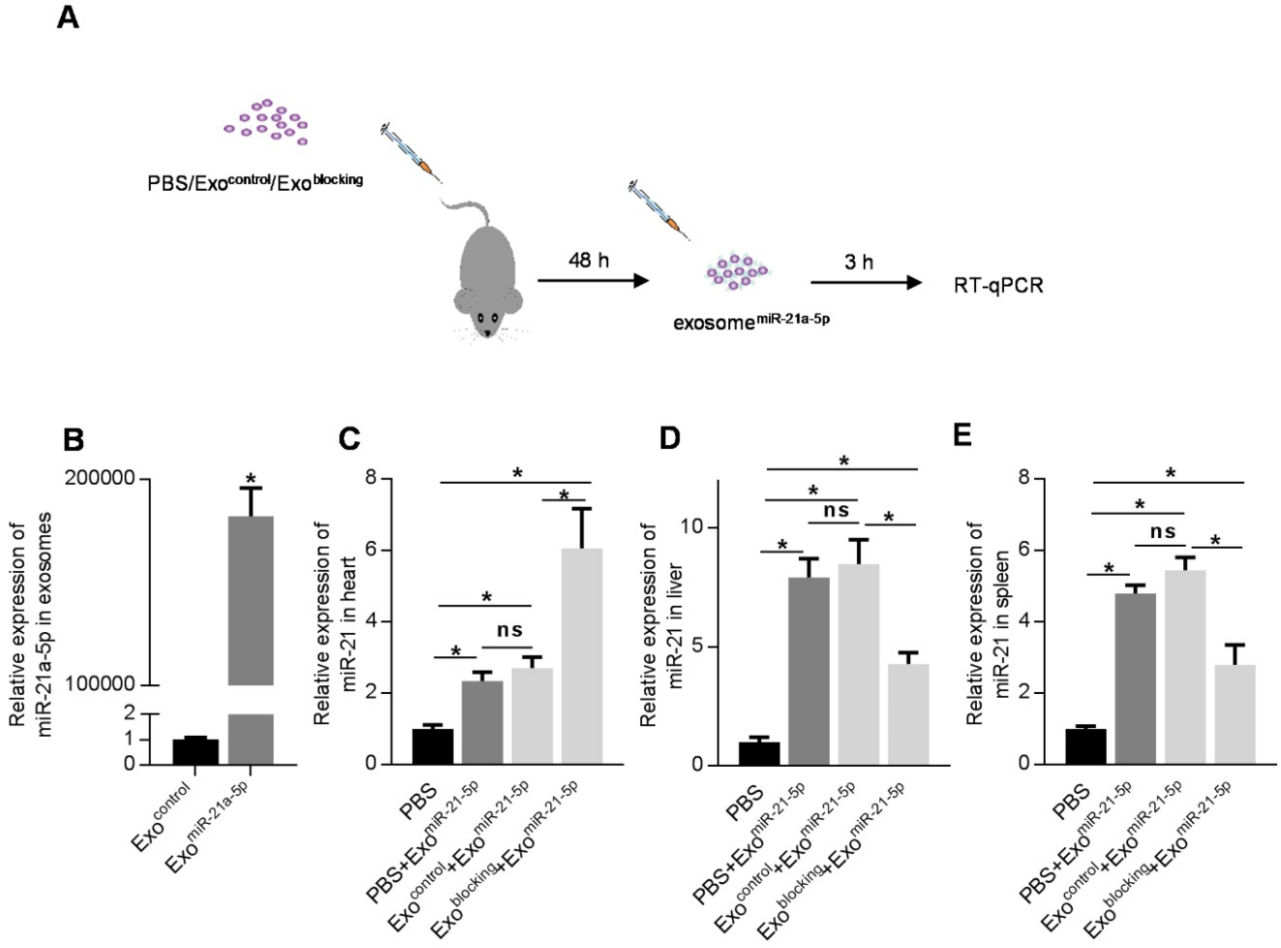
** Pre-injection of exosome^blocking^ efficiently delivers exosomal miR-21a-5p into the heart.** (A) Schematic diagram of the experimental procedure. Mice treated with PBS/exosome^control^/exosome^blocking^ were subsequently injected with exosomes^miR-21a-5p^, followed by qPCR analysis of target of interest in various tissues. (B) qPCR analysis of loading efficiency of miR-21a-5p into exosomes. (C-E) qPCR analysis of delivery efficiency of miR-21a-5p in the heart (C), liver (D), and spleen (E). U6 served as an internal control and data are expressed as mean ± SEM. n=5. *, p < 0.05; ns, no significance.

To further confirm the efficacy of the prior injection strategy of exosome^blocking^ in drug delivery into organs other than spleen and liver, doxorubicin-induced cardiotoxicity model was used. Mice were treated with 5 mg/kg/week doxorubicin for 4 weeks. Consistent with previous studies 17-19, doxorubicin induced significant apoptosis and fibrosis in the heart (Figure S6A, B), as well as decreased cardiac function (Figure S6C, D). To develop therapeutic exosomes, miR-21a-5p was encapsulated into the exosomes, as it was reported to be a potent cardiac protective miRNA in multiple studies 20-24. The miR-21a-5p mimic was electroporated into the exosomes (Figure 5A, Figure 5B) 25-27 and the engineered exosomes were named exosome^therapeutic^. There was enhanced localization of exosomes in the heart by exosome^blocking^ (Figure 4) and prior injection of exosome^blocking^ significantly improved miR-21a-5p delivery into the heart with about 4-fold enhancement compared with the controls (Figure 5C). Additionally, prior injection of exosome^blocking^ markedly decreased miR-21a-5p delivery into the liver and spleen (Figure 5D, E).

**Figure 6:**
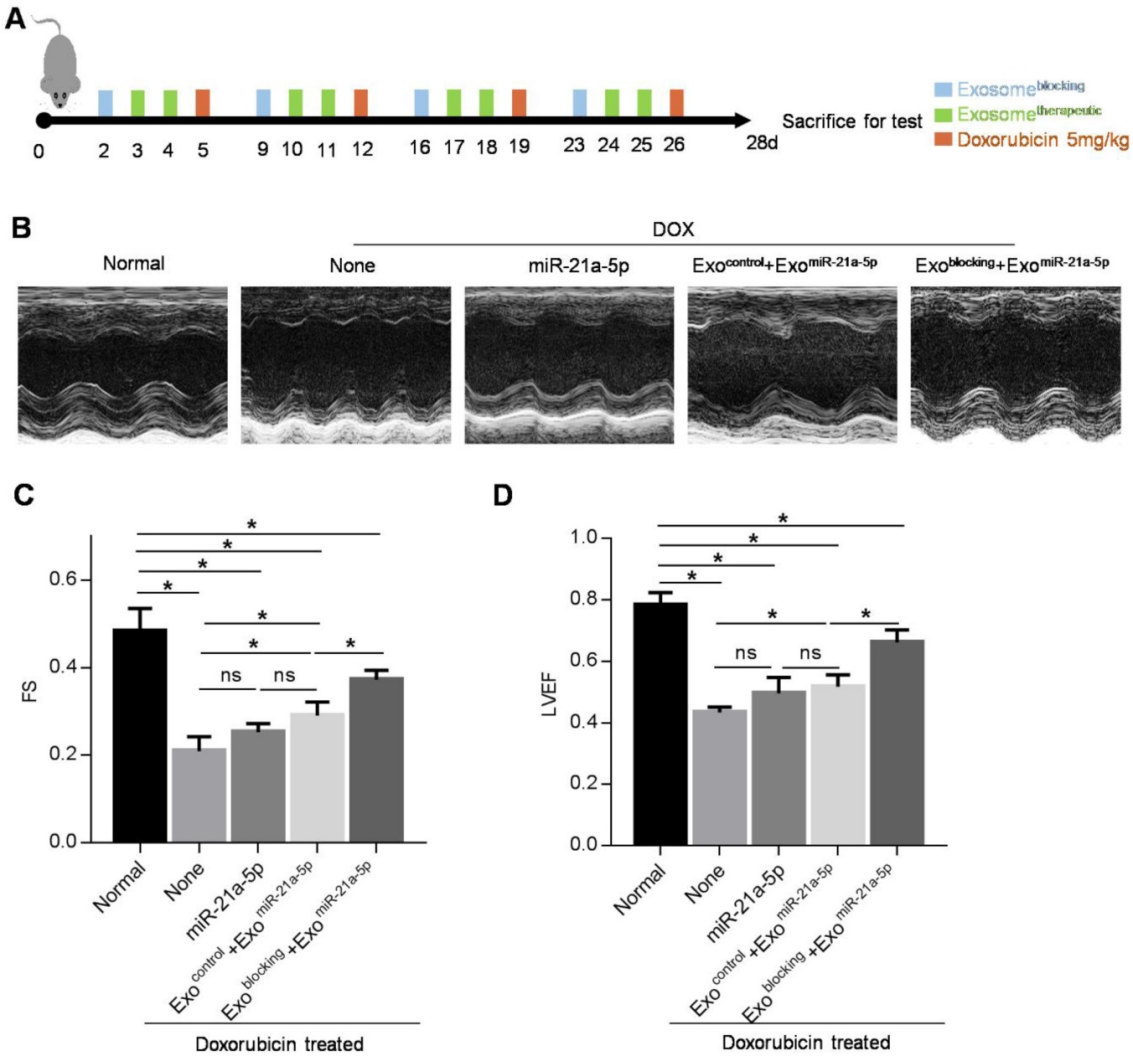
** Pre-injection of exosome^blocking^ improves therapeutic effect of exosome^therapeutic^ on cardiac damage caused by doxorubicin.** (A) Schematic diagram of the experimental procedure. Treatment time points of doxorubicin, exosome^blocking^ and exosome^therapeutic^ are indicated. (B) Representative M-mode echocardiographic images in various groups treated as indicated. n=5 mice per group. (C-D) Left ventricular ejection fraction (LVEF) (C) and fractional shortening (FS) (D) of the hearts in the groups as indicated above. Data are expressed as mean ± SEM of 5 mice per group. *, p < 0.05; ns, no significance.

**Figure 7:**
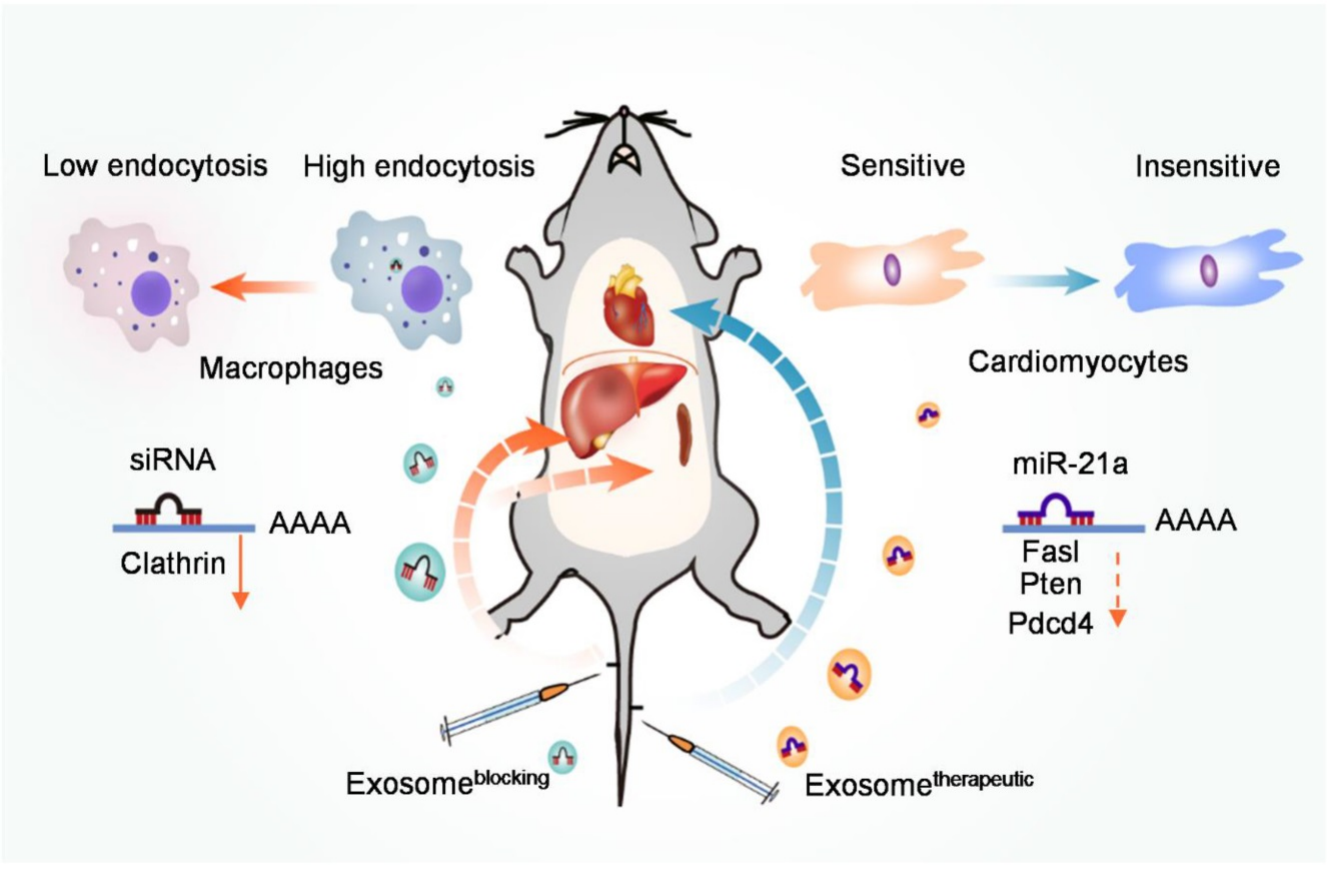
** Schematic illustration of the study.** Prior injection of exosome^blocking^ significantly blocks the MPS, allowing efficient delivery of therapeutic exosomes to the heart in the second injection, and could be used to improve therapeutic efficacy and prevent chemotherapy-induced cardiotoxicity.

Next, we explored whether prior injection of exosome^blocking^ could boost the beneficial effect of therapeutic exosomes in the DOX-induced cardiotoxicity model. Mice were injected with PBS/exosome^control^/exosome^blocking^ 3 days before DOX treatment, and then exosome^therapeutic^ were injected 1 day before DOX treatment. After 4 consecutive weeks of 5 mg/kg doxorubicin treatment, mice were subjected to echocardiography analysis (Figure 6A). Treatment of exosome^therapeutic^ alone or together with the prior injection of exosome^control^ had minimal cardiac protective function (Figure 6B-D) and a slight increase of miR-21a-5p was observed (Figure 5C). However, treatment of exosome^therapeutic^ together with prior injection of exosome^blocking^ significantly rescued the cardiac function damaged by DOX (Figure 6B-D). Thus, our study demonstrated that prior injection of exosome^blocking^ substantially blocked the MPS and allowed efficient delivery of the second therapeutic injection to targeted tissues. This strategy could be applied not only for preventing chemotherapy-induced cardiotoxicity but also for other diseases (Figure 7).

## Discussion

In the present study, we, for the first time, found that Cltc plays a significant role in exosome uptake by the MPS. Inhibition of Cltc by prior injection of exosomes loaded with siRNA against Cltc significantly blocked the endocytosis by MPS in the spleen and liver, resulting in increased exosome delivery in the following exosome injections to other organs, such as the heart. As a proof-of-concept study, we confirmed that the prior blockade strategy with siCltc-modified exosomes significantly improved the protective effect of the miR-21-loaded exosomes in doxorubicin-induced myocardial toxicity model.

Previous studies have suggested that exosomes could be a great carrier to deliver nucleic acids and other drugs for therapeutic purposes 28. However, a major obstacle to the exosome-based treatments is the preferential uptake by the MPS in the spleen and liver, restricting their clinical application 29. Some studies have used topical injections to improve the delivery efficiency of exosomes 21, whereas other studies focused on engineering exosomes with targeting peptides on the surface 25, 30, 31. However, these strategies resulted in limited, if any, improvement. In this study, we employed a novel strategy of prior blocking of the endocytosis function of macrophages in a noninvasive and effective way to improve the exosome delivery efficiency to organs other than the MPS.

Uptake of exosomes by macrophages proceeds via more than one route, such as Cltc-mediated endocytosis (CME), micropinocytosis, phagocytosis, and plasma membrane fusion 32. It has been well established that phagocytosis plays a dominant role in the uptake of large nanoparticles 33, 34. However, it remains controversial whether phagocytosis or endocytosis plays a leading role in the uptake of exosomes by macrophages. Accumulating experimental evidence suggests that exosomes are usually taken up into cells via endocytosis. It has been reported that dynasore and chlorpromazine, specific inhibitors of Cltc-dependent endocytosis (CDE), can greatly suppress exosome uptake by macrophages 35, 36. Also, dynamin2 is a GTPase necessary for CDE 37, and its inhibition prevents exosome internalization significantly 38.

In the current study, we found the expression of Cltc to be much higher in RAW264.7 than in NRCMS and HUVECs, suggesting an active CDE in the MPS. In the heart tissue, on the other hand, the expression of caveolin1 was much higher than Cltc, suggesting that different cells might have different mechanisms for exosome uptake. We also found that inhibition of Cltc by siRNA significantly blocked the uptake of exosomes by MPS in the follow-up injections. Since the blocking effect sustained for a maximum of 72 h, encapsulation of long half-life siRNAs in the blocking exosomes warrants further exploration. Furthermore, we could not exclude other pathways involved in the uptake of exosomes by the MPS, as some exosomes could still be taken up by macrophages even when Cltc expression was suppressed. Phagocytosis is a receptor-dependent phenomenon that is employed to internalize particles with a diameter of at least 85 nm, suggesting that further modification of the therapeutic exosomes with CD47 might further improve the efficacy of MPS evasion 39, 40.

Herein, as a proof of concept study, we explored the therapeutic effects of the prior blockade strategy in the doxorubicin-induced cardiotoxicity model. Doxorubicin, a broad-spectrum anticancer drug, is mainly used for the treatment of malignant hematological diseases and is highly cardiotoxic 41. Although liposomes and other synthetic nanoparticles as drug carriers have been developed to circumvent this problem, dose-dependent cardiotoxicity remains the leading cause of the decreased clinical use of doxorubicin 42. Emerging studies have shown that miR-21 could prevent cardiomyocytes from apoptosis by suppressing the expression of PDCD4, KBTBD7, FasL, and PTEN 22. Tong et.al have reported that miR-21 could protect cardiomyocytes from apoptosis by regulating the expression of BTG2 43. Similarly, Song and colleagues found that localized injection of exosomes encapsulating miR-21 effectively protected cardiac function after myocardial infarction 21. We explored the therapeutic effects of the prior blockade of the MPS in the doxorubicin-induced cardiotoxicity model. Our study demonstrated that injection of miR-21-loaded exosomes could significantly protect myocardium from apoptosis when the exosomes were efficiently delivered into the heart by first employing the blockade strategy.

In conclusion, our study has revealed that Cltc plays a critical role in exosome uptake by the macrophages. Prior injection of exosomes loaded with siRNA against Cltc could significantly block the endocytic function of the MPS in the spleen and liver, and thus significantly improve the *in vivo* distribution of the subsequently injected therapeutic exosomes in other targeted organs (Figure 7).

## Supplementary Material

Supplementary figures and tables.
