# Supplementary material for: Mononuclear phagocyte system blockade improves therapeutic exosome delivery to the myocardium
Source: Theranostics. 2020 Jan 1;10(1):218–30. doi: 10.7150/thno.38198 (PMC6929612; doi:10.7150/thno.38198)
Supplement: Supplementary file 1 — Supplementary figures and tables. [file thnov10p0218s1.pdf]

Supplementary data

Mononuclear phagocyte system blockade improves therapeutic exosome delivery to the myocardium

Zhuo Wan[1,#], Lianbi Zhao[2,#], Fan Lu[3,#] , Xiaotong Gao[1] ,Yan Dong[1], Yingxin Zhao[1],

Mengying Wei[3] , Guodong Yang[3], Changyang Xing[2,*] Li Liu[1,*]

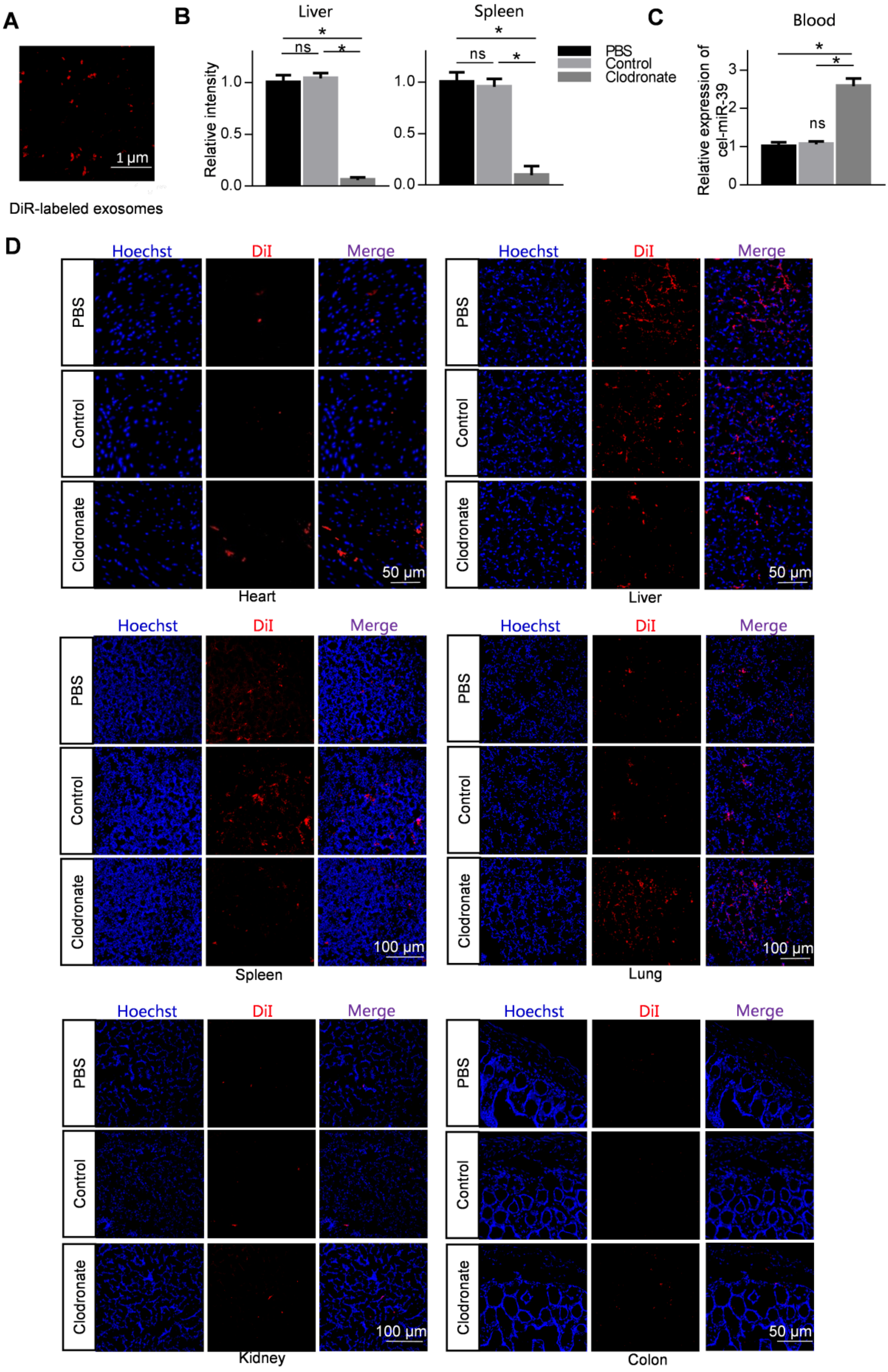

A
1 μm
DiR-labeled exosomes
B
Liver
Spleen
Relative intensity
ns
*
1.0
0.5
0.0
PBS
Control
Clodronate
C
Blood
Relative expression of cel-miR-39
3
2
1
0
D
Hoechst
DiI
Merge
PBS
Control
Clodronate
50 μm
100 μm
Heart
Liver
Spleen
Lung
Kidney
Colon

Figure S1 Macrophage depletion increases the distribution of exosomes in organs other than liver and spleen. (A) Representative fluorescence image of the DiR-labeled exosomes. Scale bar = 1 μm. (B) Relative intensity of DiR labeled exosomes localization in liver and spleen (corresponding to Figure 2E). (C) qPCR analysis of expression of cel-miR-39 in blood in mice treated with PBS/liposome/Clodronate liposome. U6 served as internal control. Data are expressed as mean ± SEM of five independent biological samples. *, $p < 0.05$. (D) Confocal images of DiI-labeled exosomes in organs of mice treated with PBS/liposome/Clodronate liposome. Data shown were representative of 5 mice in each group.

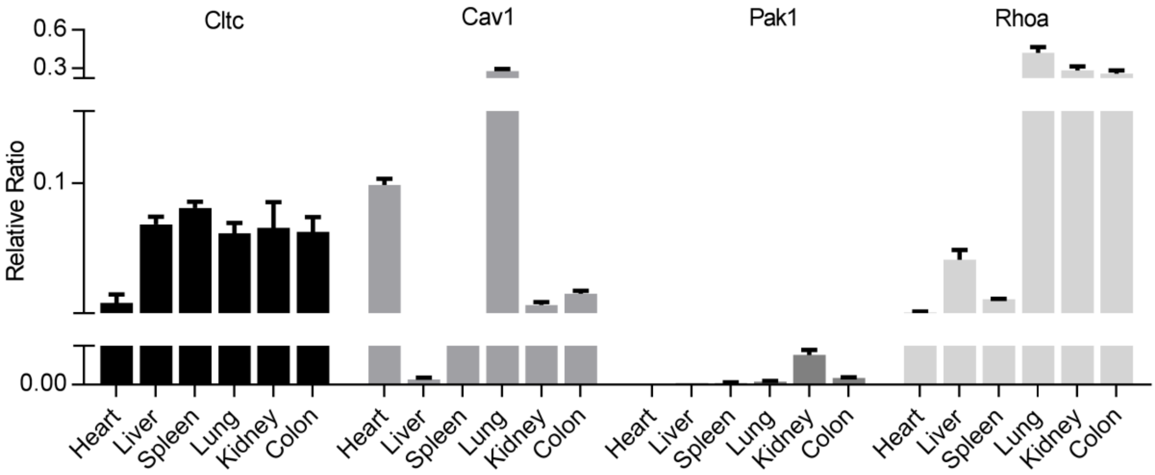

Figure S2 Expression of Cltc in different organs. qPCR analysis of the expression of endocytosis related genes (Clathrin, Caveolin1, Pak1, RhoA) in different organs in mice. GAPDH served as internal control. All the data are expressed as mean ± SEM of 5 mice.

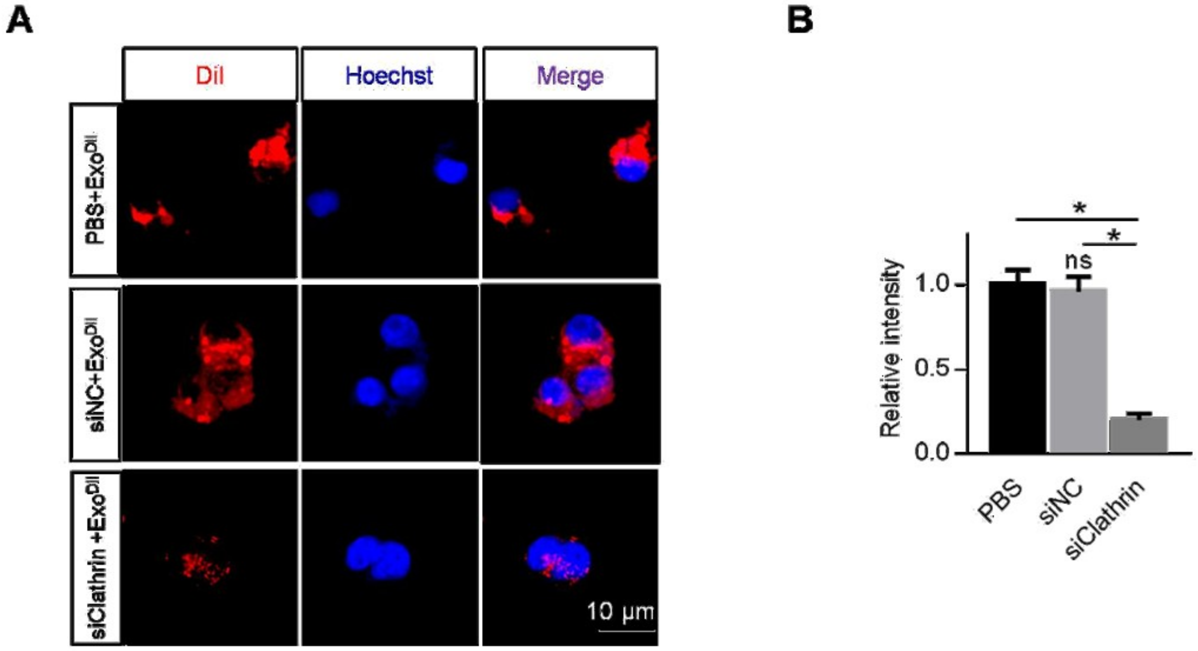

Figure S3 Clathrin knockdown inhibits phagocytosis in macrophages. (A) Fluorescence microscope analysis of endocytosis of the DiI-labeled exosomes in RAW264.7 with indicated treatments. DiI-labeled exosomes in red and Hoechst in blue. Scale bar = 10 μm. (B) Quantitative data of Figure S3A.

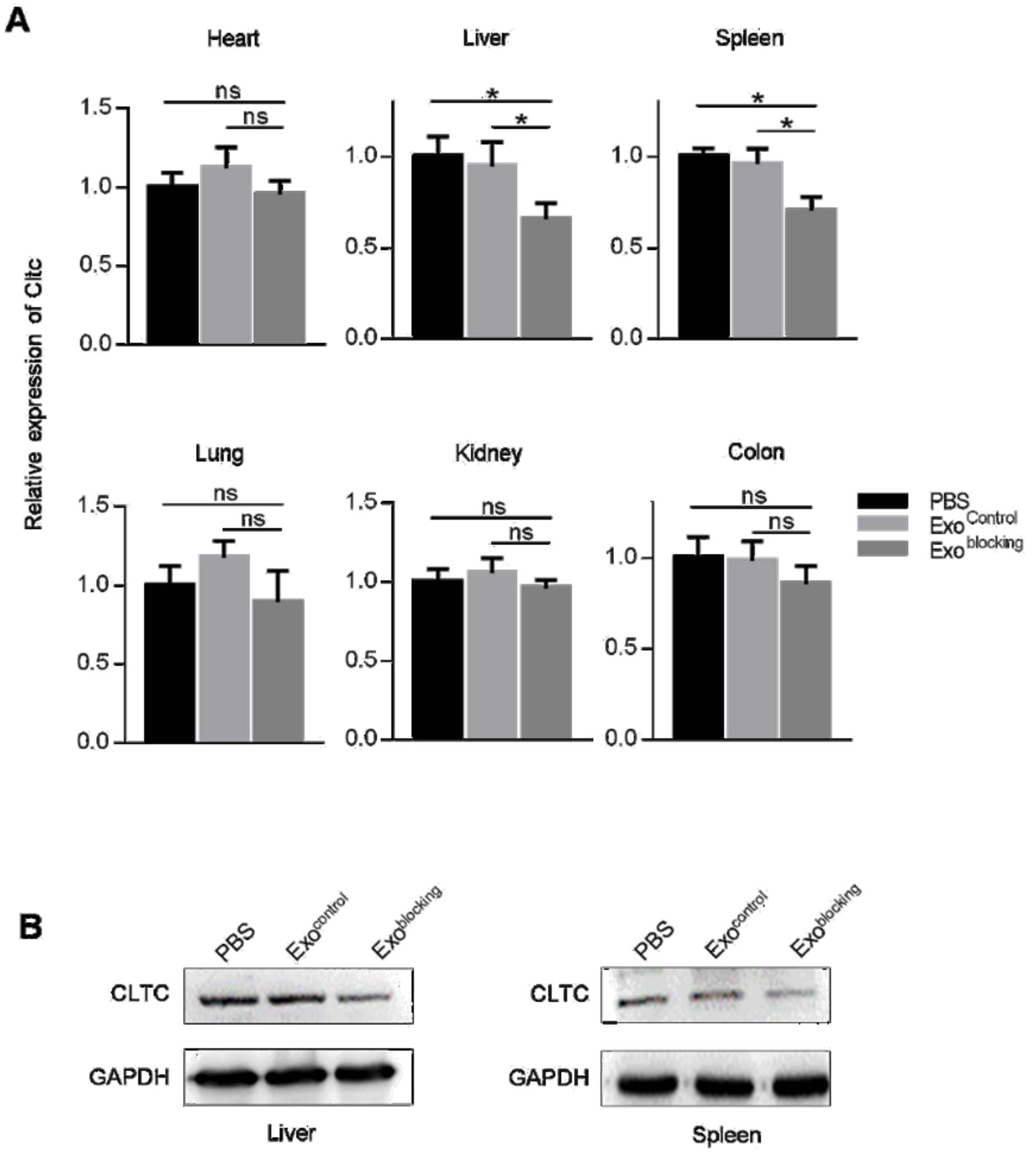

Figure S4 Exosome^{blocking} downregulates the expression of Clathrin in liver and spleen. (A) qPCR analysis of Clathrin in different organs of mice treated as indicated. GAPDH served as internal control. Data are expressed as mean ± SEM of 5 mice per group. *, $p < 0.05$. (B) Western blot analysis of Clathrin expression in liver and spleen in mice treated as indicated. GAPDH served as loading control.

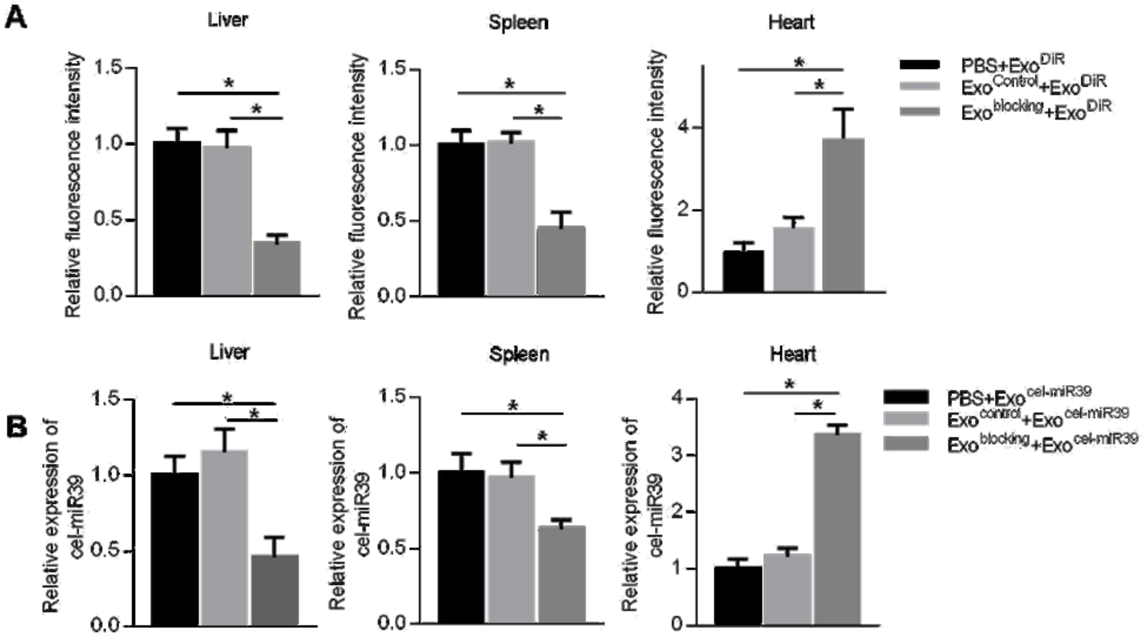

Figure S5 Prior injection of exosome^{blocking} improves the cardiomyocyte localization of later injected exosomes. (A) Quantitative analysis of the fluorescence intensity of Figure 4D. (B) qPCR analysis of the cel-miR-39 delivered in different organs. U6 served as internal control. Data are expressed as mean ± SEM. n=5 in each group. *, p<0.05.

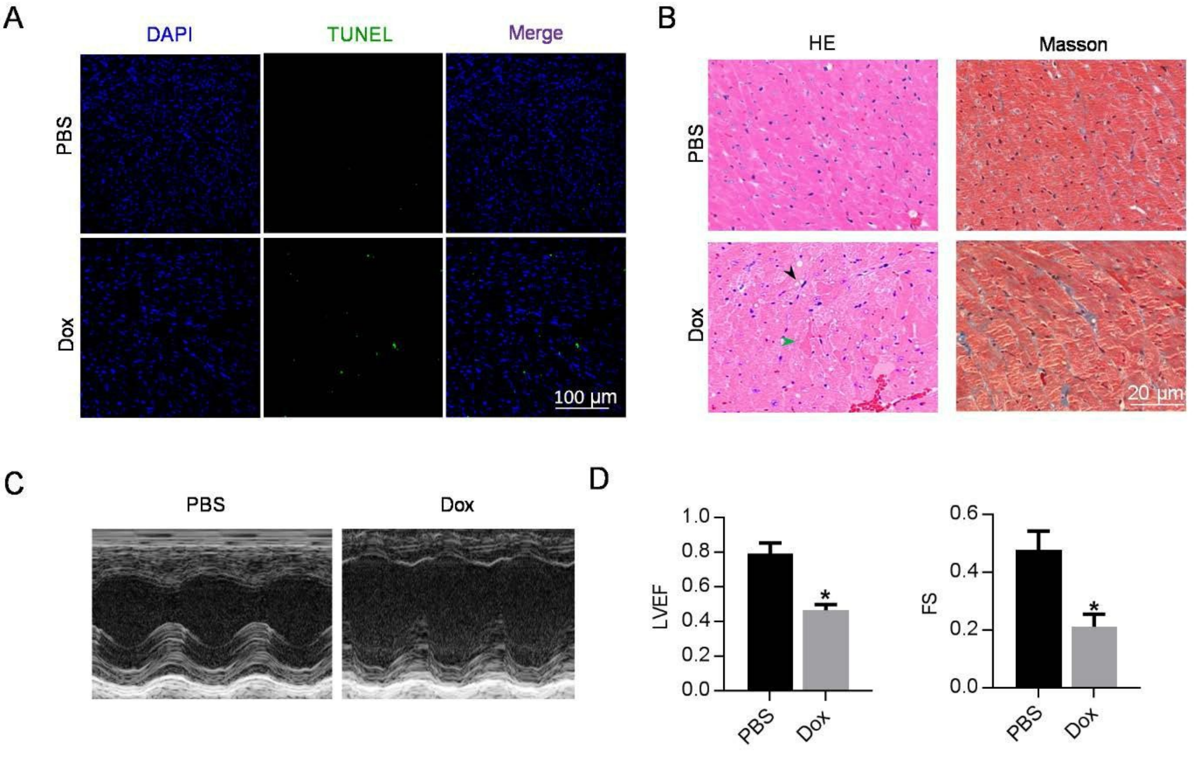

Figure S6 Cardiotoxicity induced by doxorubicin. (A) Representative TUNEL staining

images of myocardium in different groups (TUNEL in green and Hoechst in blue). Mice were treated with PBS or doxorubicin (5 mg/kg/week) for 4 weeks. n=5 per group. (B) Representative HE and Masson staining images of myocardium in mice treated same as above. Black arrowhead indicates the vacuolation of cardiomyocytes, while green arrowhead shows deeply stained cytoplasm of damaged cardiomyocytes. (C) Representative M-mode echocardiography images in mice treated as above. (D) Statistical analysis of left ventricular ejection fraction (LVEF) and fractional shortening of the ventricular minor semi axis (FS). Data are expressed as mean ± SEM. n=5. *$p < 0.05$.

Table S1 Primers used in the study.

Gene	Forward primer	Reverse primer
human CLTC	ATTCTGCCAATTCGTTTTCAGGA	GCTTTCAGTGCAATTACTTTGCT
mouse Cltc	CTTTGGCACAGGGATAGGAAAT	GCTGATCTTTTTGCTTTCGGTT
rat Cltc	CATCATCGTCTGTGACCGCT	ACTGGCCACGTTCATAAGCA
human CAV1	GCGACCCTAAACACCTCAAC	ATGCCGTCAAAACTGTGTGTC
mouse Cav1	TGCAGAACCAGAAGGGACAC	TTGGGATGCCGAAGATCGTA
rat Cav1	TTTACCGCTTGCTGTCTACCA	TGATGCACGGTACAACTGCC
human RHOA	AGCCTGTGGAAAGACATGCTT	TCAAACACTGTGGGCACATAC
mouse RhoA	AGCTTGTGGTAAGACATGCTTG	GTGTCCCATAAAGCCAACTCTAC
rat RhoA	ATTGAAGTGGACGGGAAGCAG	AGGCACCCCGACTTTTTCTT
human PAK1	CAGCCCCTCCGATGAGAAATA	CAAAACCGACATGAATTGTGTGT
mouse Pak1	CACCAGCACTATGATTGGAGC	ATTCCCGTAAACTCCCCTGTG
rat Pak1	GGCTTAGACGTCCAGGACAAA	TCGATAGAACCGGTCCTTCTTTT
human GAPDH	GGAGCGAGATCCCTCCAAAAT	GGCTGTTGTCATACTTCTCATGG
mouse Gapdh	AGGTCGGTGTGAACGGATTTG	GGGGTCGTTGATGGCAACA
rno-Gapdh	GGTGCTGAGTATGTCGTGGAG	GCGGAGATGATGACCCTTTT
mmu-miR-21a-5p	TAGCTTATCAGACTGATGTTGA	Provided in the kit
cel-miR-39	TCACCGGGTGTAAATCAGCTTG	Provided in the kit
U6	GGATGACACGCAAATTCGTGAA	Provided in the kit

Table S2 siRNAs/miRNAs used in the study.

Name	sense	antisense
siControl	UUCUCCGAACGUGUCACGUTT	ACGUGACACGUUCGGAGAATT
siClathrin	GCUCAUCAAUGUUUGCAAUTT	AUUGCAAACAUUGAUGAGCTT
mmu-miR-21a-5p	UAGCUUAUCAGACUGAUGUUGA	AACAUCAGUCUGAUAAGCUAUU
cel-miR-39	UCACCGGGUGUAAAUCAGCUUG	AGCUGAUUUACACCCGGUGAUU
